# Supplementary material for: Key Sweet Potato Viruses in Fujian Province and Their Distribution, Harmfulness, and Implications in China
Source: Curr Issues Mol Biol. 2025 Apr 1;47(4):242. doi: 10.3390/cimb47040242 (PMC12026454; doi:10.3390/cimb47040242)
Supplement: Supplementary file 1 [file cimb-47-00242-s001.zip › cimb-3528507-supplementary.pdf]

Table S1. Specific primers used for determination of sweet potato virus.

Virus species	Primer name	Primer sequence (5′→3′)	PCR product size /bp	Annealing temperature/°C
SPVG	SPG-F	GTATGAAGACTCTCTGACAAATTTTG	1191	69.7
SPVC	SPC-F	GTGAGAAAYCTATGCGCTCTGTT	836	70.9
SPFMV	SPF-F	GGATTAYGGTGTTGACGACACA	589	70.4
SPV2	SP2-F	CGTACATTGAAAAGAGAAACAGGATA	369	69.7
	SPFCG2-R	TCGGGACTGAARGAYACGAATTTAA		70.9
SPFMV	SPFMV-F	GGACGAGACACTAGCAA	703	63.5
	SPFMV-R	TTCTTCTTGCGTGGAGACGT		68.2
SPLV	SPLV-F	GCCGAYGAAACCATCMTCGAT	1076	55.6
	SPLV-R	GTCTCYGGTATAAGACAAAAAG		52.1
SPVMV	SPVMV-F	GCGAATTCTCAAGCACTGAAGAAAC	1015	57.9
	SPVMV-R	CTCTCGAGTTACTGCACACCTCTCATT		61.1
SPCFV	SPCFV-F	GAAGAGTAGCYCTGARGTRAARG	1109	54.2
	SPCFV-R	AGAAATCRYAAGACAACRRTCCC		52.4
CMV	CMV-F	ATGGACAAATCTRAATCAACCAG	657	52.4
	CMV-R	TCARACTGGGAGCACYCCWGAYGT		59.6
SPCSV	P1	GGTCATGATGATTCCGATCTT	702	66.9
	P2	CCATGGATTCAATTCAAATTTAGAG		67.6
SPCSV	P3	AGTCGGCATAGATTGGATTC	598	66.2
	P4	CTGCCTTTGAGATTGCTCTT		66.2
SPCSV	P5	GACGGKGGTACKATGAARGTCC	431	74.1
	P6	GGCTCACAAACHGAYTTCATAAACAT		71
SPMMV	SPMMV-A	ACCGGGAGATGGCGATGAA	268	69.7
	SPMMV-B2	CACGTGATACATRGCGCTTCTTA		70.9
SPLCV	SPLCV-CPF	TAAGTCGACATGACAGGGCGAATTCCC GTTTC	783	59.3
	SPLCV-*CP*R	GCGCTCGAGTTAATTATTATGTGAATCA T		50.8
SPLCV	SPLCV-F	GCCTGATTACATAAACTAAATTGA	420	65.3
	SPLCV-R	TTAAGA ACGCAATATTCTGTG		63
SPPV	rt-badB-F	TCGGCAGTAACAGACTACTTGG	147	71.3
	rt-badB-R	TCTGCTTAT CATCTCCGTTGG		68.9
SPSMV-1	rt-mas1-F	AGGCTTTCTATGCGAGTTGTGG	130	71.3
	rt-mas1-R	CACGTATTT GGGAAGTTCTGG		68.9
TLCV	AV494	GCCYATRTAYAGRAAGCCMAG	576	69.9
	AC1048	GGRTTDGARGCATGHGTACATG		70.7

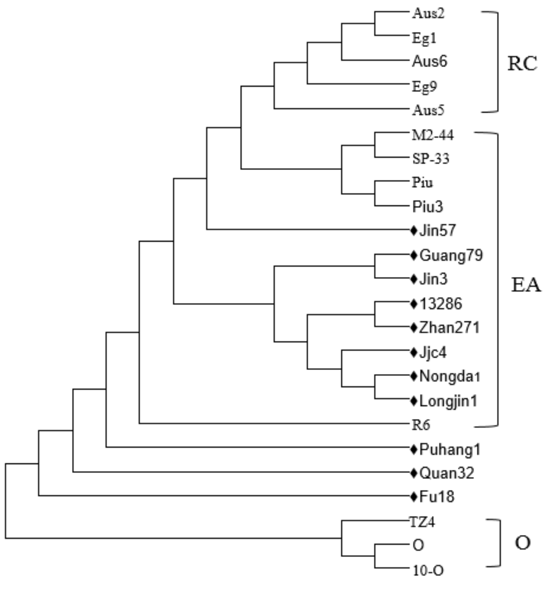

Figure S1. Neighbor-Joining phylogenetic dendrogram of SPFMV isolates based on gene nucleotide sequences.

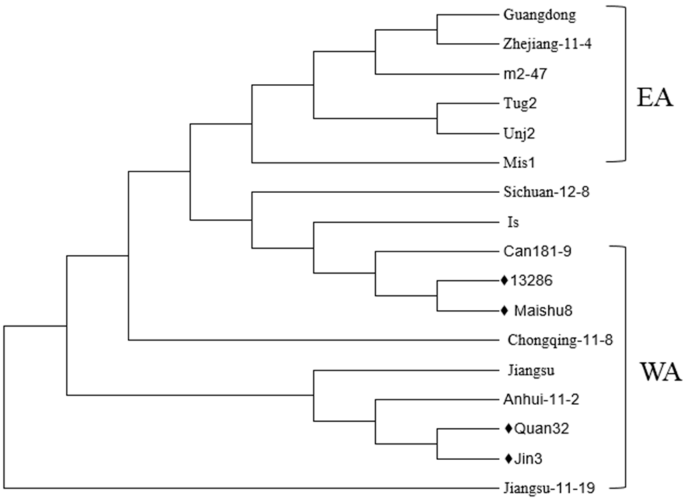

Figure S2. Neighbor-Joining phylogenetic dendrogram of SPCSV isolates based on gene nucleotide sequences.

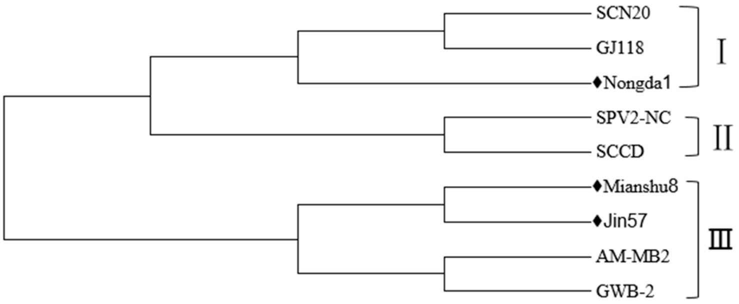

Figure S3. Neighbor-Joining phylogenetic dendrogram of SPV2 isolates based on gene nucleotide sequences.

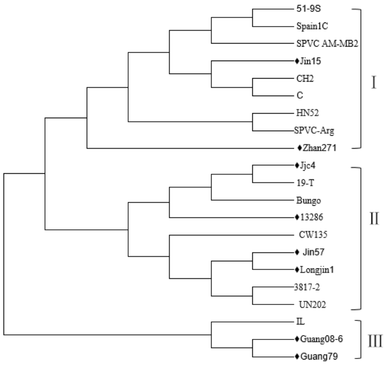

Figure S4. Neighbor-Joining phylogenetic dendrogram of SPVC isolates based on gene nucleotide sequences.

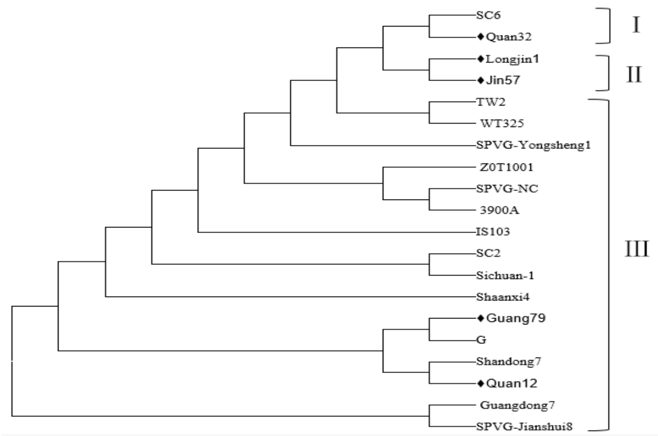

Figure S5. Neighbor-Joining phylogenetic dendrograms of SPVG isolates based on gene nucleotide sequences.

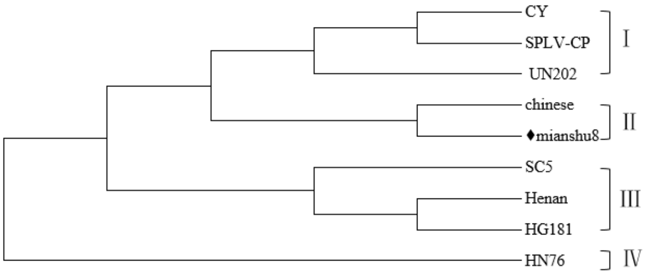

Figure S6. Neighbor-Joining phylogenetic dendrogram of SPLV isolates based on gene nucleotide sequences.

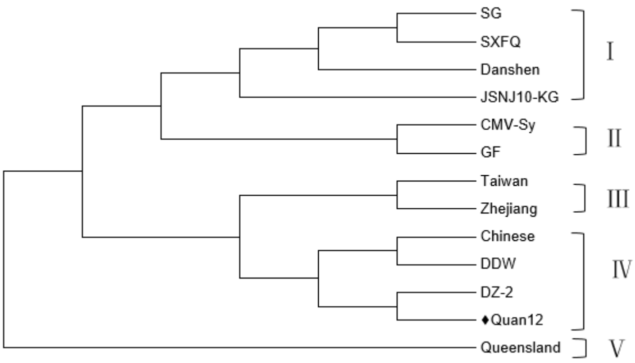

Figure S7. Neighbor-Joining phylogenetic dendrogram of CMV isolates based on gene nucleotide sequences.

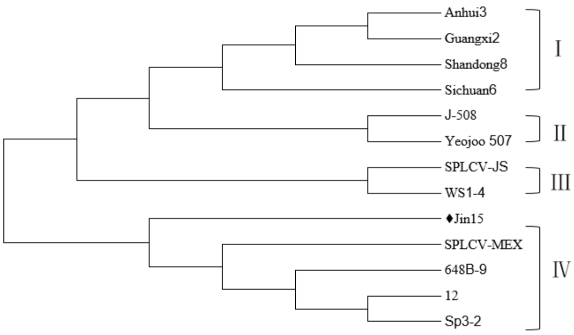

Figure S8. Neighbor-Joining phylogenetic dendrogram of SPLCV isolates based on gene nucleotide sequences.

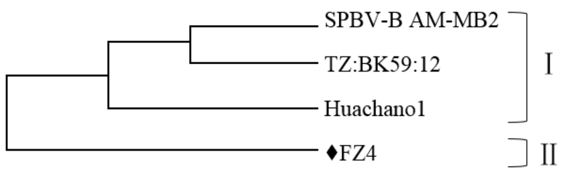

Figure S9. Neighbor-Joining phylogenetic dendrogram of SPPV isolates based on gene nucleotide sequences.

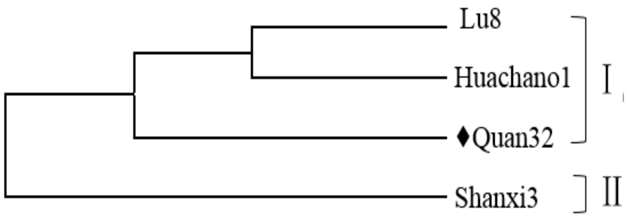

Figure S10. Neighbor-Joining phylogenetic dendrogram of SPSMV-1 isolates based on gene nucleotide sequences.

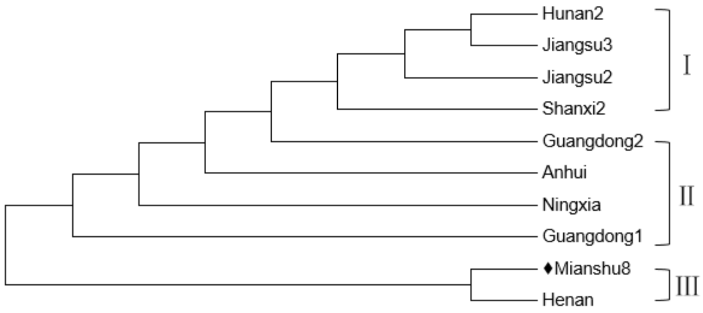

Figure S11. Neighbor-Joining phylogenetic dendrogram of SPVMV isolates based on gene nucleotide sequences.
